# Microbiota succession during aerobic stability of maize silage inoculated with *Lentilactobacillus buchneri* NCIMB 40788 and *Lentilactobacillus hilgardii* CNCM‐I‐4785

**DOI:** 10.1002/mbo3.1153

**Published:** 2020-12-24

**Authors:** Pascal Drouin, Julien Tremblay, Justin Renaud, Emmanuelle Apper

**Affiliations:** ^1^ Lallemand Specialities Milwaukee WI USA; ^2^ National Research Council of Canada, Energy, Mining, and Environment Montréal QC Canada; ^3^ London Research and Development Center Agriculture and Agri‐food Canada London ON Canada; ^4^ Lallemand SAS Blagnac France

**Keywords:** aerobic stability, corn, inoculant, lactic acid bacteria, microbiota, silage

## Abstract

Aerobic deterioration of silage following feeding out is responsible for the deterioration of its quality. Inoculation of silage with lactic acid bacteria is one strategy to limit these effects. A trial was performed using whole‐plant corn ensiled in bag silo, and forage was inoculated with *Lentilactobacillus buchneri* NCIMB 40788 (*Lactobacillus buchneri*) and *Lentilactobacillus hilgardii* CNCM‐I‐4785 (*Lactobacillus hilgardii*) or not (Control silage). After 159 days of fermentation, the silos were opened and the silage was sampled at 24‐h intervals during a 10‐day aerobic stability assay to measure pH, the fermentation profile, mycotoxins, and microbial and fungal populations. In inoculated silage, lactic acid concentrations and pH remained stable during the aerobic phase and higher microorganism alpha‐diversity was observed. Treated silage was characterized by a high abundance of *Saccharomycetes* and maintenance of *Lactobacillus* throughout the aerobic stability assay. The high aerobic stability of the inoculated silage contrasted with the decrease in lactic acid contents and the increase in pH observed in the Control silage, concomitantly with an increase in lactate‐assimilating yeast (*Pichia* and *Issatchenkia*), and in *Acetobacter* and *Paenibacillus* OTUs. Remarkably, *Penicillium* and roquefortine C were detected in this silage by day 8 following exposure to air. Our study highlighted the fact that the use of *L. buchneri* with *L. hilgardii* modified the consequences of exposure to air by maintaining higher microbial diversity, avoiding the dominance of a few bacteria, and preventing fungi from having a detrimental effect on silage quality.

## Graphical abstract

Aerobic deterioration of silage following feeding out is responsible for important quality losses on farms. Inoculation with a combination of *Lentilactobacillus buchneri* and *Lentilactobacillus hilgardii* modified the consequence of exposure to air and maintained higher microbial diversity, avoiding spoiling microorganisms detrimental effect on the silage.

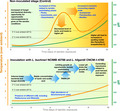

## 1 INTRODUCTION

Ensiling is a way to preserve forage based on the production of organic acids under anaerobic conditions. Ensiling consists of different successive phases, the final one being the feed‐out period. Air diffusion inside the pile of silage at this phase leads to aerobic deterioration of silage and depends on several factors, including humidity, density, permeability, porosity, and temperature of the stored forage (Pitt & Muck, 1993). Two mechanisms will allow oxygen to enter the silage mass: diffusion and volumetric flow (Pahlow et al., 2003) and both result in material losses. Losses due to the diffusion of oxygen inside the silage have been estimated at 120 g kg^−1^ of fresh matter in bunker and clamp silos but can exceed 300 g kg^−1^ for the crop ensiled close to the surface (Wilkinson & Fenlon, 2013). It is estimated that losses of dry matter during aerobic deterioration are more important than the losses occurring in the initial aerobic phase and the main fermentation phase (Wilkinson & Davies, 2013). In addition to the important impact on fresh matter losses, aerobic deterioration can reduce the nutritional quality of the silage due to the catabolism of silage fermentation products, leading to lower feed intake, contamination by mycotoxins, and increased health‐related issues for the animals (Gallo et al., 2015; Pitt & Muck, 1993).

Oxygen entering the silage allows microorganisms to use optimal biochemical pathways and dormant cells may be reactivated, leading to major biochemical changes (Wilkinson & Davies, 2013). Spoiling will start with the degradation of organic acids by yeasts (Pahlow et al., 2003) and occasionally in the case of whole‐crop silages, by acetic acid bacteria (AAB) (Dolci et al., 2011). Losses of organic acids due to respiration increase the pH, thereby allowing other microorganisms, including enterobacteria, AAB, and *Bacillus* to grow, with counts reported to be as high as 1 × 109 CFU × g^−1^ (Jonsson, 1991). Degradation of more complex compounds follows the oxidation of soluble substrates. The increased biochemical activity linked to the development of all these microorganisms triggers a rise in temperature inside the silage mass, which is the most noticeable sign of the process. Subsequently, the development of less challenging physicochemical conditions following the catabolism of organic acids can trigger the development of molds. These organisms significantly reduce the nutritional quality of the feed by degrading carbohydrates, fiber, and protein. Synthesis of mycotoxins may occur simultaneously, as recently reported for aflatoxins (Cavallarin et al., 2011; Ferrero, Prencipe, et al., 2019). Microbial diversity following exposure to air has recently been studied using next‐generation sequencing, but several days after the opening of the silos. Testing aerobic deterioration of small grain silages (barley, oat, and triticale) the bacterial and fungal core microbiome composition was reported after 14 days of exposure (Dunière et al., 2017).

Developing strategies to limit aerobic deterioration is important for optimizing feed storage, livestock performance, and farm profitability. Inoculation of the forage by lactic acid bacteria improves both the fermentation process and aerobic stability (Muck et al., 2018). The selection of highly competitive strains (Carvalho et al., 2020) can improve the production of lactic acid as well as that of other organic acids able to inhibit the proliferation of undesirable fungi (Guimarães et al., 2018). Driehuis et al. (1999) observed that strains of *Lentilactobacillus buchneri* (*Lactobacillus buchneri* (Zheng et al., 2020)) were able to degrade lactic acid into acetic acid and 1,2‐propanediol (Oude Elferink et al., 2001), which could then be metabolized into propionic acid (Krooneman et al., 2002). Since both acetate and propionate are strong yeast inhibitors (Guaragnella et al., 2010; Lourenco et al., 2011), these modifications positively improve the aerobic stability of silage. More recently, co‐inoculation with *L. buchneri* NCIMB 40788 and *Lentilactobacillus hilgardii* CNCM‐I‐4785 (*Lactobacillus hilgardii*) was reported to increase the stability of different silages (Ferrero et al., 2019; Silva et al., 2020). While microbial dynamics during fermentation was recently characterized in corn silage inoculated with these two microorganisms (Drouin et al., 2019), little research has been performed to characterize microbial succession and mycotoxin production in inoculated vs uninoculated silages during the feed‐out phase (Hu et al., 2018; Liu et al., 2019). Thus, the objective of this study was to investigate the positive impact of inoculation of whole‐plant maize silage with *L. buchneri* NCIMB 40788 and *L. hilgardii* CNCM‐I‐4785 on microbial succession following aerobic exposure.

## 2 MATERIAL AND METHODS

### 2.1 Corn silage

Whole‐plant corn (hybrid Mycogen TMF2Q419) was harvested 95 days after sowing (Chazy, NY, USA: N44° 53.421′ W73° 28.103′) at a mean dry matter content of 383 g kg^−1^ The forage was harvested with a John Deere 8800 series harvester and chopped to a theoretical fragment length of 12.7 mm using a kernel processor unit (John Deere KernelStar). The corn was harvested in the field following a randomized five‐block design to minimize field‐based variations.

The forage was transported to the ensiling site for inoculation using a Dohrman DE‐25 applicator mounted on the bagging system (Ag‐Bag Systems G6170, Wisconsin, USA). The applicator nozzles were mounted on the filling ramp of the bagger and adjusted to deliver 800 ml min^−1^ of either water (Control) or a mix of *Lentilactobacillus buchneri* NCIMB 40788 (*Lactobacillus buchneri*) and *Lentilactobacillus hilgardii* CNCM‐I‐4785 (*Lactobacillus hilgardii*) (LB + LH), each set at 2 × 10^5^ CFU g^−1^ of fresh forage. Individual replication (*n* = 5/treatment) corresponded to two metric tons of forage separated by one ton of uninoculated forage as a spacer. The treated region was marked with spray paint on the outside of the bag. Each replication corresponded to one of the five randomized field‐based blocks of the crop, which was used for both treatments. To reduce contamination, LB + LH was inoculated and bagged after the control by turning the applicator on and off in turn to manage the spacers. Both treatments were completed within 2 h. The two bags were placed next to one another to guarantee similar storage conditions. The bag silos were 3.5 m in width.

The bagged silage was opened after 159 days of fermentation by removing the plastic cover along the complete length of the bags. Silage from the outer core was removed and silage samples were taken from the central core (1.5 m from the side) of each silage replication using a loader. One set of (250 g) samples was frozen and used for the analysis of the fermentation parameters. The second set of (250 g) samples was used for microbial counts and the determination of the dry matter in a forced‐air oven (at 55°C for 48–72 h). Lastly, the third set of (20 g) samples was frozen at −80°C for DNA extraction and high‐throughput sequencing.

### 2.2 Aerobic stability assay

The aerobic stability assay was performed according to the Honig method (1990) by collecting silage from the central core of the bag silage for all five replications. For each experimental unit, two one‐kg layers of silage were loosely packed in a 7‐L container and covered with a layer of cheese‐cloth. Air‐permeable lids were used to cover the container and reduce moisture loss. A temperature probe (TMC6‐HD, Onset Computer, Massachusetts, USA) was positioned between each one‐kg layer. The temperature probes were connected to data loggers (UX120‐006M, Onset Computer, Massachusetts, USA). Ambient temperature was recorded using the same probes inserted in a bucket filled with perlite to buffer rapid temperature changes. All 10 buckets (5 replications × 2 treatments) were incubated in an incubation chamber set at 20°C.

### 2.3 Experimental design of the aerobic stability succession trial

Additional samples were collected from the central core to perform an aerobic stability succession trial at the same time as the AS assay. For this purpose, 60 experimental units were collected from the central core for replications 1, 2, and 3 of the bag silos in each treatment, according to a design based on 10 experimental units per replication. These additional AS experimental units were prepared using the same technique as described above and were incubated in the same temperature‐controlled room. The experimental units were randomly distributed as one per period of AS, equal to day 1, 2, 3, 4, 5, 6, 7, 8, 9, and 10 of exposure to air, totaling three replications per treatment per day of aerobic exposure (2 treatments × 3 replications × 10 sampling periods = 60). At 24‐h intervals during the AS succession trial, about 1 kg of the top layer of silage was removed and two 250 g samples were collected per unit from the middle of the six containers (2 treatments × 3 replications = 6 per 24 h period) for further analysis and the remaining silage was discarded. One set of samples was used for chemical analysis and the second for DNA isolation and high‐throughput sequencing.

### 2.4 Microbial counts

Upon sampling (day 0 to day 10), for the microbial counts, 20 g samples of silage were refrigerated and transported as rapidly as possible to the laboratory for analysis. The samples were suspended with 180 ml of NaCl buffer (0.85%) for two 60‐s periods in a Stomacher 400 paddle blender mixer (Seaward, UK). Serial dilutions were then performed using the same NaCl buffer. Lactic acid bacteria were enumerated using De Man‐Rogosa‐Sharpe agar plates (Oxoid—Thermo Scientific, Hampshire, UK) containing 100 µg L^−1^ cycloheximide (Sigma‐Aldrich, USA). Yeast and molds were enumerated on malt extract agar plates (Oxoid—Thermo Scientific, Hampshire, UK) containing 2 g L^−1^ of Rose Bengal (Fisher Scientific, USA), streptomycin at 100 µg L^−1^, and neomycin at 50 µg L^−1^ (Sigma‐Aldrich, USA).

### 2.5 Chemical analysis

The nutritional characteristics (ADF, NDF, starch, crude protein, soluble protein, and ethanol soluble carbohydrates) of samples of fresh forage were analyzed at Cumberland Valley Analytical Services (Waynesboro, PA, USA) using wet chemistry methods (https://www.foragelab.com/Resources/Lab‐Procedures). Volatile fatty acids (VFA), lactic acid, ethanol, and pH were also analyzed at Cumberland Valley Analytical Services. Briefly, each fermented silage sample was mixed, and a 25 g wet sample was taken and diluted with 200 ml deionized water. After incubation for 2 h in the refrigerator, the sample mixture was then blended for 2 min and filtered through a coarse (20–25 µm particle retention) filter paper. For VFA quantification and ethanol, 3 ml of extract was filtered through a 0.2 µm filter membrane, and a 1.0 µl subsample was injected into a Perkin Elmer AutoSystem gas chromatograph (Model 710, Perkin Elmer, USA) equipped with a Restek column packed with Stabilwax‐DA (Restek, USA). For lactic acid quantification, a 1:1 ratio of extract to deionized water was placed in a YSI 2700 Select Biochemistry Analyzer to determine lactic acid. pH was measured with a Mettler DL12 Titrator (Mettler‐Toledo, USA) using 0.1 N NaOH to a pH of 6.5.

### 2.6 DNA isolation

The DNA isolation and purification protocols were adapted from the methodology proposed by Romero et al. (2018) and Zhou et al. (2016). DNA was extracted from replications 1, 2, and 3. Five grams of silage samples were weighed in a 50 ml conical centrifugation tube and suspended in 10 ml of sterile deionized water. The samples were then sonicated in a Branson model 8800 ultrasonic water bath at 40 kHz for 5 min and vortexed for 1 min. A 3‐ml aliquot of the corn silage suspension was centrifuged, and the pellet was transferred to tubes containing beads in the PowerLyzer Soil DNA Isolation Kit (MoBio Laboratories, Carlsbad, NM, USA). Microbial lysis was optimized by 2 min of mechanical lysis in a MixerMill 400 (Retsch, Inc., Haan, Germany) at a speed of 15 cycles per second. DNA isolation then proceeded according to the manufacturer's protocol. The concentration of DNA was measured on a spectrophotometer (Nanodrop Technology, Cambridge, UK) and quality was measured by agarose gel electrophoresis (1% agarose). The concentration of DNA was standardized at 2 ng μL^−1^ for all samples.

### 2.7 High‐throughput sequencing and bioinformatics analysis

For amplicon sequencing, the libraries were prepared according to the Illumina 16S Metagenomic Sequencing Library Preparation guide (Part # 15044223 Rev. B), except that a Qiagen HotStar MasterMix (Toronto, Ontario, Canada) was used for the first PCR (amplicon PCR) and half the volume of reagents was used for the second PCR (index PCR). This protocol includes a PCR cleanup step that uses AMPure XP beads to purify amplicons from free primers and primer dimers. The template‐specific primers were as follows (without the overhang adapter sequence): 515F (5′‐GTG CCA GCM GCC GCG GTA A‐3′) and 806R (5′‐GGA CTA CHV GGG TWT CTA AT‐3′) for 16S amplification from the V4 hypervariable region (Caporaso et al., 2011), and with ITS region 1 specific primers ITS1F (5′‐CTT GGT CAT TTA GAG GAA GTA A‐3′) and 58A2R (5′‐CTG CGT TCT TCA TCG AT‐3′) for the amplification of fungi (Yergeau et al., 2017). The amplicon PCR reaction was carried out for 30 cycles with annealing temperatures of 55°C for 16S and 45°C for ITS. Diluted pooled samples were loaded on an Illumina MiSeq and sequenced using a 500‐cycle MiSeq Reagent Kit v2 (San Diego, California, USA, adapted from Yergeau et al. (2017)). The average size of the amplicon sequences was 293 bp for the 16S regions and 276 bp for the ITS regions.

Sequencing data were analyzed using AmpliconTagger (Tremblay & Yergeau, 2019). Briefly, raw reads were scanned for sequencing adapters and PhiX spike‐in sequences, and the remaining reads were merged using their common overlapping part with FLASH (Magoč & Salzberg, 2011). Primer sequences were removed from merged sequences and the remaining sequences were filtered for quality such that sequences with an average quality (Phred) score of less than 27 or one or more undefined bases, (N) or more than 10 bases with a quality score of less than 15 were discarded. The remaining sequences were clustered at 100% identity and then clustered/denoized at 99% identity (DNACLUST v3, (Ghodsi et al., 2011)). Clusters with abundances lower than three reads were discarded. The remaining clusters were scanned for chimeras with the VSEARCH version of UCHIME de novo and UCHIME reference (Edgar et al., 2011; Rognes et al., 2016) and clustered at 97% (DNACLUST) to form the final clusters/OTUs. A global read count summary is provided in Table A1. Bacterial and fungal OTUs were then assigned a taxonomic lineage with the RDP classifier (Wang et al., 2007) using the AmpliconTagger 16S and ITS training sets, respectively (Tremblay, 2019; https://doi.org/10.5281/zenodo.3560149). The RDP classifier attributes a score (0 to 1) to each taxonomic depth of each OTU. Each taxonomic depth with a score of >= 0.5 was retained to reconstruct the final lineage. Taxonomic lineages were combined with the cluster abundance matrix obtained above to generate a raw OTU table. From that raw OTU table, an OTU table containing bacterial organisms only was generated. Five hundred 1000 read rarefactions were then performed on the latter OTU table and the average number of reads of each OTU of each sample was then computed to obtain a consensus rarefied OTU table. A multiple sequence alignment was then obtained by aligning OTU sequences on a Greengenes core reference alignment (DeSantis et al., 2006) using the PyNAST v1.2.2 aligner (Caporaso et al., 2010). Alignments were filtered to keep only the hypervariable region of the alignment. A phylogenetic tree was built from that alignment with FastTree v2.1.10 (Price et al., 2010). Alpha (observed species) diversity metrics and taxonomic summaries were then computed using the QIIME v1.9.1 software suite (Caporaso et al., 2010; Kuczynski et al., 2011) using the consensus rarefied OTU table and phylogenetic tree (i.e., to build a UniFrac distance matrix). Analysis of the ITS amplicons was performed similarly, but the alignment was performed using the Silva core reference database (Quast et al., 2012).

The 16S and ITS rDNA raw reads from the microbiota analyses have been deposited at the NCBI BioProject repository under study accession number PRJNA595554.

### 2.8 Non‐targeted mycotoxin analysis

Samples were extracted based on the multi‐mycotoxin method of Sulyok et al. (2006). Briefly, 0.2 ± 0.02 g of ground silage was extracted with 1 ml of 79/20/1 (v/v/v) acetonitrile/water/acetic acid. The solutions were first vortexed for 30 s, sonicated at 35°C for 30 min, and finally shaken on a thermomixer (35°C, 1400 rpm) for 30 min. The samples were then centrifuged and 400 μl extracts were removed. The extracts were diluted 1:1 with 20/79/1 (v/v/v) acetonitrile/water/acetic acid prior to LC/MS analysis. The samples were analyzed using a non‐targeted, data‐independent acquisition‐screening method as described in Renaud et al. (Renaud & Sumarah, 2016) to allow the identification of a multitude of mycotoxins and fungal secondary metabolites by accurate mass, retention time as well as MS/MS spectra. Briefly, the samples were analyzed in a non‐quantitative way that allowed the identifiable features of all the ionizable compounds present to be recorded. The resulting data contain high‐resolution accurate mass information of all precursors and product ions as well as the retention times. Analytes that were putatively first detected by accurate mass to charge ratio (*m*/*z*) (±3 ppm) and retention time were confirmed by matching the product ions *m*/*z* (±3 ppm) with standard spectra deposited in the MassBank Europe spectral library (https://massbank.eu/MassBank). Semi‐quantification was then performed by running a calibration curve containing the detected mycotoxins with a matrix‐matched calibration solution.

### 2.9 Statistical analysis

The effect of the day of exposure to air on the chemical parameters, mycotoxins, and the differences between treatments was analyzed by a two‐way ANOVA using R version 3.6.1 (R Core Team, 2020). The treatments and the day of aerobic exposure were considered as fixed factors. Interactions between treatments and days were tested. The following parameters were individually tested by the model: pH, lactic acid, acetic acid, ethanol, and the ratio of lactic acid to acetic acid. If the interactions were significant, a pairwise comparison was performed using one Kruskal–Wallis rank test per individual sampling period. For the mycotoxins, values below the threshold of detection were replaced by one half of the detection limit. Aerobic stability was tested by performing a Kaplan–Meier analysis using the time needed to reach the 2°C and 3°C thresholds above the ambient temperature. The maximum temperature was also recorded. Similarly, differences in the abundance of specific OTUs were tested using a Kruskal–Wallis rank test within an individual day. Significant differences were *p* < 0.05.

According to our experimental design, differentially abundant OTUs (DAO) were assessed with edgeR (v3.19.7) using its GLM approach (Chen et al., 2016) with OTU table raw count matrix as input. OTU having a logFC (log foldchange) ratio equal to or higher than 1.5 and false discovery rate (FDR) < 0.05 were considered as differentially abundant between each aerobic stability period. The log transformation of the FC (foldchange) and CPM (counts per million) values were used to generate histograms using in‐house scripts, including ggplot functions.

## 3 RESULTS

### 3.1 Fermentation parameters

Upon opening of the bag silos, the dry matter level was, respectively, 382 and 399 g kg^−1^ for Control and LB + LH (*p* = 0.018). The content of ADF (*p* = 0.586), NDF (*p* = 0.115) and crude proteins (*p* = 0.885) were of 19.86, 33.44, 7.18 and of 19.42, 32.62, 7.08% DM for Control and LB + LH, respectively. The chemical parameters measured after fermentation and during the feed‐out phase are listed in Tables 1 and 2. The interaction between treatments and time was significant for all the chemical parameters (i.e., pH, lactic acid, lactic:total VFA ratio, ethanol, and total VFA) except acetic acid for which only a significant treatment effect was observed (Table 1). The interaction showed that the parameters tested in the Control silages (pH, lactic acid) did not remain constant with time, whereas in the LB + LH silages, they did not change significantly (Table 2). For acetic acid, the concentration upon opening was 1.01 g kg^−1^ DM for Control silage and 1.67 g kg^−1^ DM for LB + LH. During aerobic exposure, they were of 1.67 (*p* = 0.107 over time) and 0.72 g kg^−1^ DM (*p* = 0.873 over time) for Control and LB + LH, respectively.

**TABLE 1:** Probability values for fermentation parameters between treatments and interaction with the time of incubation

	pH	Lactic acid	Acetic acid	Lactic/VFA	Ethanol	VFA total	NH_3_‐CPE
Treatment	<0.001	<0.001	<0.001	<0.001	0.756	0.018	<0.001
Time (day of AS)	<0.001	<0.001	0.276	<0.001	<0.001	<0.001	0.197
Treatment × Time	<0.001	<0.001	0.406	<0.001	<0.001	0.014	0.003

**TABLE 2:** Changes in fermentation parameter every 24 h of the aerobic stability assay

Time	pH			Lactic acid (g kg^−1^ DM)		
(day AS)	Control	LB + LH	*p*	Control	LB + LH	*p*
0	3.87^a^	3.97	<0.001^b^	34.4	26.4	<0.001
1	4.07	4.08	0.050	21.6	29.2	0.050
2	4.06	4.07	0.507	19.7	26.1	0.127
3	4.08	4.06	0.513	17.0	24.0	0.050
4	4.09	4.07	0.369	16.3	22.1	0.081
5	4.10	4.06	0.246	16.8	25.6	0.006
6	4.67	4.09	0.050	11.7	25.9	0.083
7	4.78	4.07	0.077	7.1	24.5	0.027
8	5.56	4.03	0.050	5.2	22.8	0.035
9	4.98	4.07	0.038*	5.4	20.5	0.026
10	5.47	4.10	0.028*	5.2	22.6	0.037

Time	Ethanol (g kg^−1^ DM)			Ratio lactic acid:VFA (%)		
(day AS)	Control	LB + LH	*p*	Control	LB + LH	*p*
0	14.00	8.80	<0.001	77.6	61.5	0.003
1	11.46	6.00	<0.001	61.4	77.6	0.109
2	11.10	7.39	0.049	57.6	78.5	0.073
3	10.30	7.79	0.127	53.7	72.8	0.074
4	8.59	6.46	0.173	51.0	78.2	0.035
5	8.62	7.45	0.073	52.8	82.0	0.002
6	4.96	5.32	0.827	33.3	85.4	0.037
7	2.96	6.12	0.506	24.4	84.1	0.019
8	1.81	5.32	0.121	15.4	75.0	0.025
9	0.42	5.89	0.004	18.2	67.7	0.025
10	0.48	4.57	0.028	14.5	72.5	0.021

Time	Ammonium (% CPE)			Total VFA (g kg^−1^ DM)		
(day AS)	Control	LB + LH	*p*	Control	LB + LH	*p*
0						
1	0.73	0.70	0.275	35.3	37.8	0.513
2	0.69	0.66	0.376	34.0	33.4	0.827
3	0.64	0.63	0.507	31.6	33.0	0.275
4	0.67	0.57	0.127	31.7	28.5	0.369
5	0.69	0.63	0.178	31.8	31.3	0.829
6	0.66	0.58	0.376	29.7	30.3	0.926
7	0.65	0.57	0.268	25.5	29.1	0.369
8	0.84	0.60	0.017	19.3	30.5	0.213
9	0.74	0.58	0.020	22.9	30.4	0.213
10	0.97	0.63	0.114	20.7	31.4	0.261

^a^ Means of triplicate analyses.^1^

Indeed, the pH was below 4.0 in the two silages upon opening, and significantly lower in Control than in LB + LH silages (−0.1 unit, Table 2). After exposure to air, it followed two trends depending on the treatment. In the control silage, the pH increased from 3.9 to 5.5 (*p* < 0.001—Kruskal–Wallis over time), while it remained relatively stable, between 3.97 and 4.10, in the LB + LH silage. In agreement with the change in pH, the lactic acid concentration was higher in the Control silage upon opening and then decreased dramatically, from 34.4 to 5.2 g kg^−1^ DM (*p* < 0.001), whereas in the LB + LH silages, it remained within a constant range (between 26.4 and 22.6 g kg^−1^ DM; *p* = 0.070). The ratio of lactic acid to total VFA followed the same pattern as lactic acid concentrations. The ethanol concentration was higher in the Control silage upon opening and decreased with time in both treatments, but the reduction was more marked in the Control silage, making the interaction significant. The acetic acid concentration was higher in the LB + LH silage but did not change significantly with time.

The mean concentration of soluble proteins over time was significantly higher in LB + LH, 35.8 g kg^−1^ DM, compared to Control silage with a mean of 30.6 g kg^−1^ DM (*p* < 0.001). An interaction was observed between the two factors (*p* = 0.012) with the decrease in soluble proteins during aerobic deterioration in the Control silage from a mean of 36.0 upon opening to 26.0 g kg^−1^ DM after 10 days of AS (*p* < 0.001). The concentration of soluble proteins in the LB + LH silage remained constant (37.7 to 35.7 g kg^−1^ DM). For Control, the decrease in a soluble protein corresponds to the increase in ammonium observed in relation to the length of aerobic exposure (Table 2). NDF, ADF, and the concentration of starch were not influenced by aerobic deterioration in either treatment, with *p* of 0.394, 0.396, and 0.805, respectively, for the length of aerobic exposure.

### 3.2 Aerobic stability

Inoculation with LB + LH significantly improved aerobic stability (*p* < 0.001), as 206.3 ± 26.2 and 128.7 ± 32.0 h were required to reach a threshold of 2°C above the ambient temperature in the LB + LH and Control silages, respectively. For a threshold limit of 3°C above ambient temperature, the LB + LH silage required 224.0 ± 21.7 h, while the Control silage required 144.7 ± 40.4 h. The maximum temperature recorded during the aerobic stability assay was 25.5 ± 1.0°C in non‐inoculated silage and 22.9 ± 1.8°C in LB + LH silage (*p* = 0.002).

**FIGURE 1:**
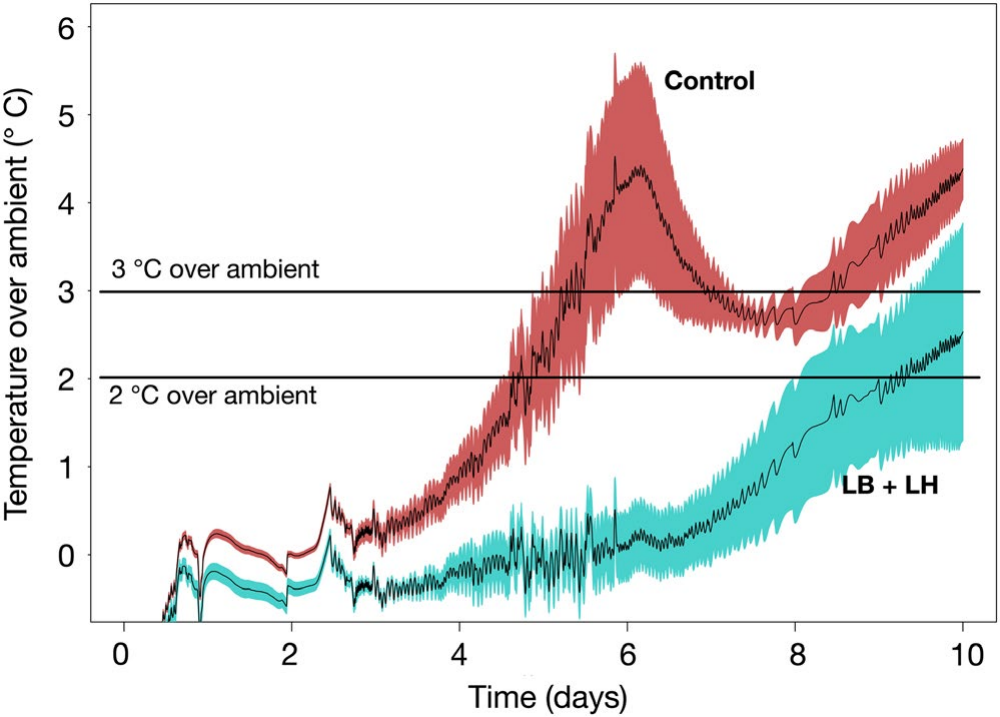
Mean temperature profile recorded over a 10‐day incubation period aerobic stability assay performed with five individual replications of silage from a bag silo. The solid line corresponds to the mean value and the colored area (red for Control—cyan for LB + LH) is the standard error of the five replications. The two horizontal lines in the figure correspond to threshold values of 2°C and 3°C above the ambient temperature.

Temperature profiles during AS are presented in Figure 1. Fresh matter losses during AS averaged 127.7 ± 23.2 g, representing 7.9% of the original weight of the silage in the Control silage while averaging 72.7 ± 5.6 g in LB + LH silage, that is 4.6% of the original weight of the silage (*p* < 0.001).

### 3.3 Microbial counts

*Upon opening*, the counts of LAB, yeast, and molds in the silos were 7.8, 2.7, and 2.3 log_10_ g^−1^ fresh forage (ff), respectively, in the Control silage and 7.4, 3.1, and below the threshold of detection level of 2 log_10_ g^−1^ ff in the LB + LH silage. The difference between treatments was only significant for the count of LAB (*p* < 0.001).

### 3.4 The bacterial population

**FIGURE 2:**
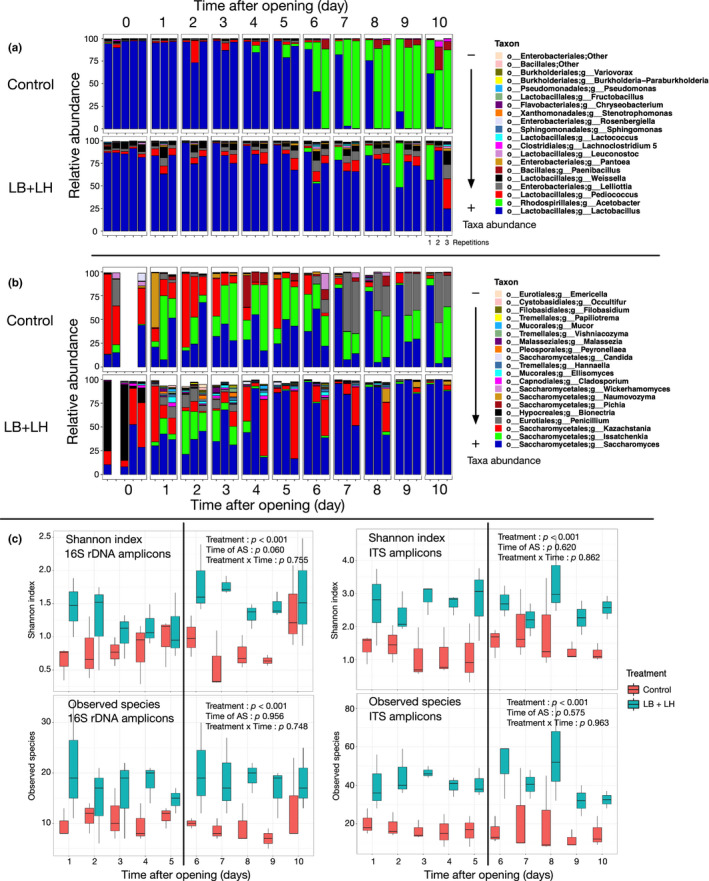
Microbiota diversity from the 16S amplicons (a) and ITS amplicons (b) for the forage upon the opening of the bag silos (incubation time 0) and results after 10 days of incubation at 20°C in the aerobic stability assay. Panel (c) presents the alpha‐diversity values from the Shannon diversity index and the operational taxonomic units (OTUs) for the 16S and ITS amplicons and each day of incubation during the aerobic stability assay. The vertical line corresponds to the time when Control samples crossed the threshold value of 2°C above the ambient temperature. The alpha‐diversity results are the mean of three replications. Control is in red and LB + LH in cyan. * represents significant difference at 95%, ** at 99%, and *** at 99.9%. For the samples collected upon the opening of the bag silos, the abundance results of all five field‐based replications are included.

Upon opening, the Shannon diversity index and the number of observed OTUs were not affected by inoculation: 0.97 ± 0.40 and 17.2 ± 5.9 in the Control silage and 1.14 ± 0.23 and 21.0 ± 6.1 in the LB + LH silage (Figure 2c). The relative abundance of OTUs related to *Lactobacillus* (updated taxonomy from the Greengenes database could not be considered at this stage) represented 95.0% in the Control silage and 86.2% in the LB + LH silage (*p* = 0.004). In the latter, a higher abundance of *Pediococcus* (2.5%) and of *Weissella* (5.0%) related OTUs (1.2% and 1.2%) was observed, with a significant difference in *Weissella* abundance compared to that in the Control silage (*p* = 0.001).

#### 3.4.1 Diversity during the aerobic stability trial

Over the 10 days, both the Shannon index and the number of observed OTUs in LB + LH silage were significantly higher, with a mean of 17.8 compared to 10.1 in the Control silage (*p* < 0.001). There was no significant effect of the length of aerobic exposure, with *p* of 0.956 and 0.060 for the Shannon index and the observed OTUs, the interaction was not significant (*p* above 0.7) in either parameter. In the Control silage, the Shannon index remained between 0.60 and 1.00 throughout the study except on day 10 when it reached 1.39. In the LB + LH silage, the Shannon index ranged between 1.0 and 1.5 throughout the study, with higher values measured on days 6, 7, and 10.

**FIGURE 3:**
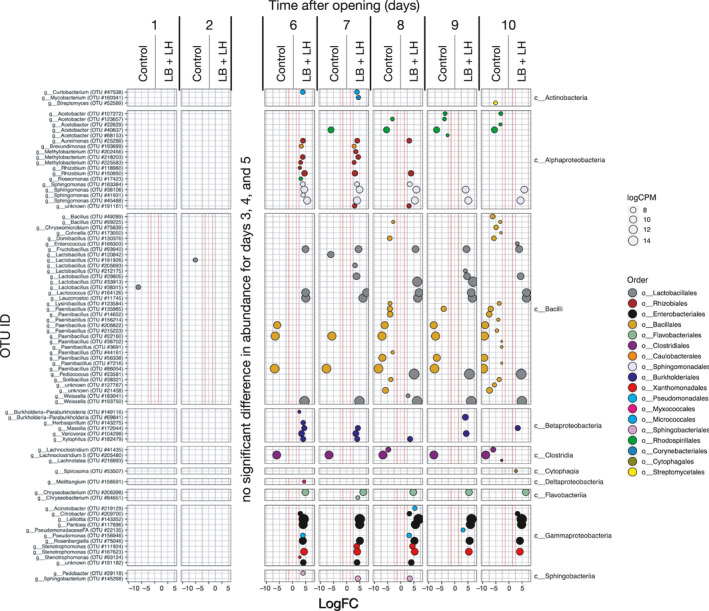
Contrast analysis of the 16S amplicon sequencing data of the samples collected at 24‐h intervals during incubation in the aerobic stability assay analyzed using the EdgeR package. Only OTUs whose abundance differed significantly between the Control and the LB + LH inoculation treatment are shown. No significant difference was observed at days 3, 4, and 5, so these days are not included in the figure. LogFC (fold changes) and logCPM (counts per million) are used for the representation. Only OTUs with a logFC ≤ 1.5 (red line in each panel) and false discovery rate (FDR) < 0.05 were considered as being differentially abundant. Color coding is based on the taxonomic classification of individual OTU.

The bacterial taxonomic profiles in the two treatments evolved differently during the incubation period of the AS assay (Figures 2 and 3). Five days after opening, the abundance of *Lactobacillus* was similar in the two treatments, with 89.1% and 82.6% in the control and LB + LH silage, respectively. Although not significantly different by day 10 due to marked variations between replications, the abundance of *Lactobacillus* decreased to 20.9% and 56.5% in the control and LB + LH silage, respectively. Strong development of OTUs related to the genus *Acetobacter* was observed in the Control silage. Starting from day 5, the relative abundance of OTUs related to *Acetobacter* increased to reach 87.6% by day 9. The increase in the abundance of *Acetobacter* OTUs was followed by an increase in OTUs related to *Paenibacillus*, reaching 11.5% by day 10. However, like *Acetobacter*, the marked variation in abundance between replications did not result in a significant difference between the two treatments. OTUs related to *Weissella* were observed in the LB + LH silage throughout the incubation period of the AS assay ranging from 3.2% to 6.3%. Although the relative abundance of *Weissella* and *Pediococcus* did not differ significantly, it dropped in the control silage while remaining more stable in the LB + LH silage.

Contrast analysis revealed no differences between silage treatments up to day 6, with only two *Lactobacillus* OTUs being significantly more abundant in the Control than in the LB + LH silage (Figure 3). From day 6 on, a time that corresponded to changes in the microbial composition (Figure 2) and the first heating period (Figure 1), higher abundances of *Burkholderiales*, *Enterobacteriales*, *Lactobacillales*, *Sphingomonadales*, *Rhizobiales*, *Pseudomonadales*, and *Xanthomonadales* related OTUs were observed in the LB + LH silage. Within the order *Lactobacillales*, the main genera involved were *Lactobacillus*, *Lactococcus*, *Leuconostoc*, and *Weissella*. Also starting from day 6, in the Control silage, higher abundances of OTUs related to *Bacillales* (*Paenibacillus* genus), *Clostridiales* (*Lachnoclostridium* genus, previously classified in the genus *Clostridium* (Yutin & Galperin, 2013)), and *Rhodosphirillales* (*Acetobacter* genus) were present than in the LB + LH silage.

### 3.5 Fungal population

Upon opening, the Shannon diversity index and the number of observed OTUs of the bagged corn silage (Figure 2b—Time 0) did not differ between the two treatments (2.05 ± 0.40 and 24.7 ± 8.6 in the Control silage and 1.64 ± 0.49 and 26.3 ± 11.8 in the LB + LH silage). Ascomycota was the dominant phylum in both the Control and LB + LH silages, with over 95% of the total abundance. In the LB + LH silage, Saccharomycetales was the most abundant order, with 63% relative abundance in the treated silage, the diversity profile differed between the five replications. Two replications had a high relative abundance of Saccharomycetales, a mean of 81%, but only in these two replications. In the other two replications of the LB + LH silage, the dominant OTU was identified as belonging to the family *Bionectriaceae* with a mean abundance of 77%. The fungal population of these last two samples was similar to the one identified in the Control silage, but with a higher abundance of *Saccharomycetaceae* (Figure 2b) and no *Trichocomaceae* (i.e., *Penicillium*). A BLAST search of an OTU identified as *Bionectria* using SYLVA (# 151859) instead provided a higher similarity score to the genus *Clonostachys* sp., which also belongs to *Bionectriaceae*. One OTU related to *Penicillium* (*Eurotiales* order) was present in one of the replications of the Control silage at 28% abundance.

#### 3.5.1 Kinetics during the aerobic stability trial

The Shannon diversity index and the number of observed OTUs did not differ significantly between sampling periods (*p* = 0.620) but on average was significantly higher in the LB + LH silage over the complete 10‐day period (*p* < 0.001). There was no interaction between time and treatment (Figure 2c).

During aerobic exposure, OTUs belonging to *Saccharomyces*, *Issatchenkia*, *Kazachstania* were dominant in the Control silage until day 6. From then on, fewer *Kazachstania* were detected, whereas the relative abundance of OTUs related to *Penicillium* increased (Figure 2b).

**FIGURE 4:**
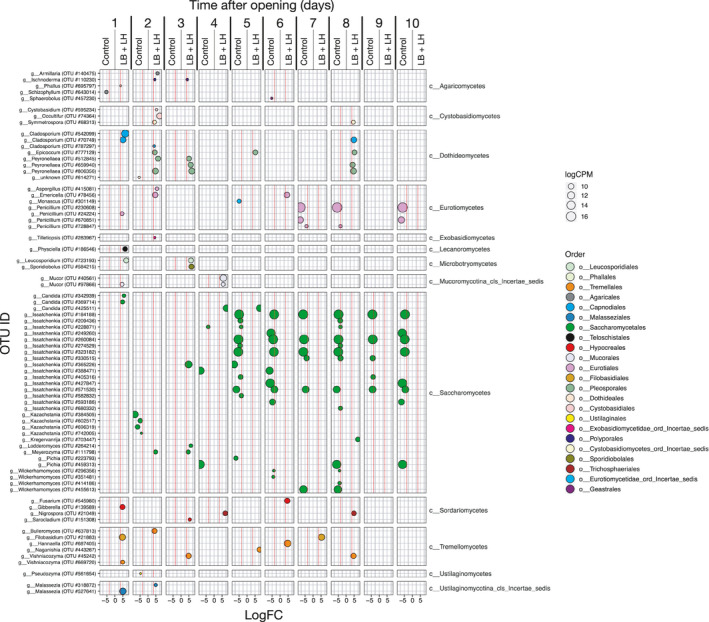
Contrast analysis of ITS amplicon sequencing data of the samples collected at 24‐h intervals during incubation in the aerobic stability assay analyzed using the EdgeR package. Only OTUs whose abundance differed significantly between the Control and the LB + LH inoculation treatments are shown. LogFC (fold changes) and logCPM (count per million) are used for the representation. Only OTUs with a logFC ≤ 1.5 (red line in each panel) and false discovery rate (FDR) value <0.05 were considered as being differentially abundant. Color coding is based on the taxonomic classification of individual OTUs.

Interestingly, in the LB + LH silage, the relative abundance of *Saccharomyces* increased with time to reach more than 80% on day 10 and with less variability between replicates, while the abundance of OTUs related to *Issatchenkia* was high from 48 h to 72 h, with 31.5% and 20.0% of the relative abundance, but from then on was never higher than 2.1% (Figure 2b). Also, it is interesting to note that several taxa were present at low relative abundances on days 1, 2, 3, notably *Pleosporales*, *Eurotiales*, *Tremellales*‐related OTUs. Contrast analysis revealed that yeasts belonging to *Malassezia* and *Filobasidium* genera, the ubiquitous mold *Cladosporium*, and fungi belonging to the orders *Pleosporales*, *Eurotiales*, *Cystobasidiales*, and *Tremellales* were significantly more abundant at days 1, 2, and 3 after opening in the LB + LH silage. Few differences were observed in the number of OTUs related to the *Saccharomycetales* order between the two treatments in the early stages of AS incubation (Figure 4). On day 4, OTUs identified as yeast *Issatchenkia* tended to be more abundant in the Control silage, with 40.4% of the total abundance (*p* = 0.080), compared to 5.3% of total abundance in the LB + LH silage. The difference in the abundance of this OTU remained in the same range in the following days. Basidiomycetous yeast belonging to the Order *Tremellales* was mostly more abundant in the LB + LH silage than in the Control silage, with *Hannaella* and *Filobasidium* becoming significantly more abundant by day 6 and 7.

In the Control silage, four OTUs related to mold *Penicillium* were significantly more abundant from day 7 on (Figure 4) and their abundance remained high up to the last day of the trial (Figure 2b). On day 5, the abundance of *Penicillium* OTUs was only 0.3% but increased to 7.6% in the following 24 h (*p* = 0.164). Abundance reached 39.2% by day 7 and then decreased to around 27%–28%. The mean daily abundance of the *Penicillium* OTUs was never higher than 0.9% in the LB + LH silage.

**FIGURE 5:**
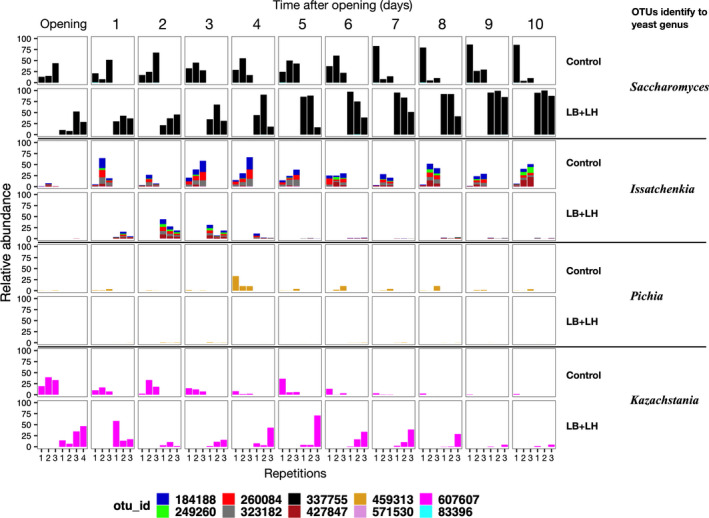
Histogram showing daily variations in OTUs related to the yeast genera *Saccharomyces*, *Issatchenkia*, *Pichia*, and *Kazachstania* in the Control and LB + LH inoculation treatments during the aerobic stability assay.

The abundance of the most abundant yeast‐related OTUs followed different trends depending on the treatment (Figure 5). The control silage was mainly characterized by the emergence of *Issatchenkia* in the first few days following exposure to air and a gradual decrease in *Kazachstania* was also observed. Also in the control silage, OTUs related to *Saccharomyces* increased slightly on the first day of exposure to air and remained stable thereafter. Conversely, a continuous increase in *Saccharomyces* was observed in the LB + LH silage, up to 94.2% on day 10, and dominated the emergence of *Issatchenkia* from day 1 to day 3. *Kazachstania* did not display a clear pattern but varied among the replications: the abundance of OTUs linked to *Kazachstania* sp. varied considerably between day 4 and day 8, reaching more than 40% in at least one of the three replications. Several OTUs linked to the yeast *Issatchenkia* were observed in both treatments (Figure 5). This may reflect a high heterogeneity of the amplicons and improved identification (Kurtzman et al., 2008).

### 3.6 Mycotoxins

The samples were screened for 84 mycotoxins and related fungal metabolites (Table A2) among which 13 were identified, including metabolites produced by *Penicillium*, *Fusarium*, and *Alternaria* (Table A3).

**FIGURE 6:**
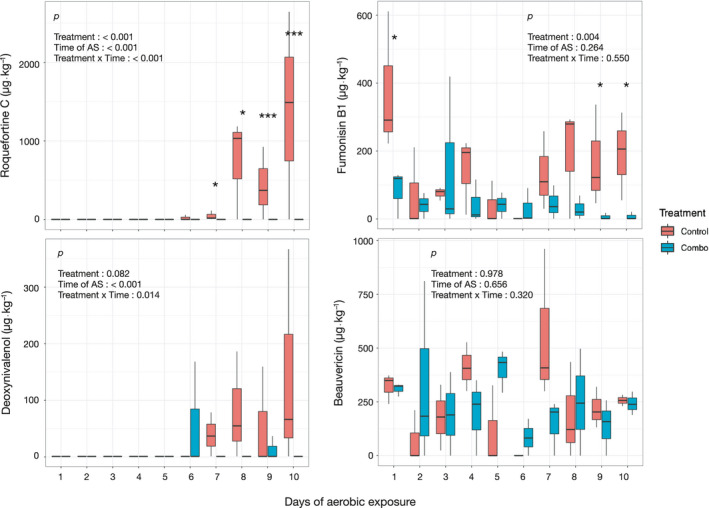
Histograms of the changes in concentrations of the mycotoxins Roquefortine C, Fumonisin B1, Deoxynivalenol, and Beauvericin every 24 hours after the start of air exposure and differences between Coinntrol and LB + LH inoculation treatments.

Three compounds observed upon the opening of silage are known to be *Fusarium* metabolites: zearalenone, fumonisin (FB), and beauvericin. The concentration of zearalenone was under 50 µg kg^−1^ of DM in the two treatments (38.3 and 47.0 µg kg^−1^ of DM in the Control and LB + LH silage, respectively, *p* = 0.827) on day 1 and did not change during exposure to air (*p* = 0.465 for the time of exposure). The concentration of FB1 was significantly higher on day 1 in the control (Figure 6), with 374.9 vs 82.9 µg kg^−1^ of DM (*p* = 0.050) in the LB + LH silage. The concentration of FB1 fluctuated with time with the mean numerically lower in the LB + LH silage, but the difference was not significant, even though there was an increase in the toxin with time in the Control versus a decrease in the LB + LH silage (Figure 6). The concentration increased to more than 170 µg kg^−1^ DM in the control silage from day 7 of incubation. The concentrations of FB2—B3 were below 10 µg kg^−1^ of DM throughout the incubation period of AS whatever the type of silage. The mean concentration of beauvericin on day 1 was 315.1 µg kg^−1^ of DM (*p* = 0.810 between treatments) and the interaction between treatment and time was not significant (*p* = 0.320).

It was not possible to detect deoxynivalenol (DON) until day 6, when the concentration increased to a mean of 144.0 µg kg^−1^ of DM by day 10 in the Control silage, although with high variability (Figure 6). In the LB + LH silage, the concentration of DON generally remained below the threshold of detection throughout the AS incubation period.

A trend similar to DON was observed for roquefortine C (Figure 6): concentration remained below the threshold of detection up to day 6, then started to increase in the Control silage, to reach 1378.0 µg kg^−1^ of DM after 10 days of incubation during AS (*p* = 0.146). Again, variability between the three replications was high. In the LB + LH silage, the concentration of roquefortine C was under the threshold of detection throughout the AS assay.

## 4 DISCUSSION

Silage exposed to air is prone to deterioration as bacteria, yeasts, and molds can be activated and oxidize different substrates, thereby affecting silage quality. The aerobic stability of silage is thus a key factor to ensure that silage provides well‐preserved nutrients for animals containing minimum levels of spores and toxins. Further development of strategies to limit problems related to aerobic deterioration is thus needed. Adding microbial inoculants during ensiling and opening periods is one possible strategy. Microbial silage additives are usually classified into two groups, homofermentative and heterofermentative. Homofermentative LAB produces lactic acid (McDonald et al., 1991), whereas heterofermentative LAB not only produce lactic acid, but also complex organic acids such as acetic acid and propionic acid (McDonald et al., 1991; Oude Elferink et al., 2001) that are strong inhibitors of yeast growth. To give an example, the heterofermentative LAB *L. buchneri* in corn silage improved AS (Dolci et al., 2011; Renaud & Sumarah, 2016), probably through a synthesis of 1,2‐propanediol and subsequent conversion to propionic acid (Oude Elferink et al., 2001). These specific properties of *L. buchneri* can be improved by co‐inoculation of a second heterofermentative LAB, *L. hilgardii* (Reis et al., 2018). The positive impact of this co‐inoculation was tested in the present study as inoculation increased the time required to reach the threshold of 2°C over ambient temperature by 77.6 h. Knowing that we analyzed the impact of the inoculation of the combination on microbial and biochemical parameters for 10 days of exposure to air.

Upon opening after 159 days of ensiling, the pH of the two types of silage was still below 4 and *Lactobacillus* related OTUs dominated, in agreement with results in the literature (Gharechahi et al., 2017; Guan et al., 2018), and reflecting the conservation process was appropriate in both cases. A recent study (with a 64‐day incubation period) using the same two lactic acid bacteria as inoculants showed that the microbiota of the inoculated corn silage was characterized by a higher abundance of *Lactobacillus* than the non‐inoculated corn silage and hosted a distinctive yeast population with a lower alpha‐diversity of the fungal related OTUs (Drouin et al., 2019). This was not confirmed in the current study, where we observed a slightly lower relative abundance of *Lactobacillus* and a higher abundance of *Weissella* in the LB + LH silage. However, the length of the fermentation period and incubation temperature differed in the two studies, and in the former study, the bag silos were stored outside. Considering the lower average temperatures encountered during the incubation of the bag silos, we would expect microbial succession to be slower than in mini‐silos stored at room temperature (Zhou et al., 2019). The presence of *Leuconostocaceae* at this late stage of ensiling is rare in corn silage, considering that upon the opening of the bag silos, pH was below 4.0, a pH to which species belonging to this family are sensitive. This level of abundance of *Weissella* sp. has sometimes been reported but in non‐inoculated silage (Keshri et al., 2018). The fungal population was mainly composed of the yeasts *Saccharomyces and Kazachstania*, but with a higher abundance of *Bionectriaceae* in some samples of LB + LH silages. *Bionectriaceae* has rarely been reported in corn silage but has been observed in sorghum before ensiling (Gonda et al., 2019). Interestingly, in this family, the *Clonostachys* related‐OTU is an epiphytic mycoparasitic fungus that can be used for biocontrol of *Fusarium graminearum* (Gimeno et al., 2019), and also has the enzymatic potential to degrade zearalenone (Ogunade et al., 2018). The heterogeneity of the fungal population in the LB + LH samples may be due to the number of fungal counts below the threshold of detection (<log_10_ of 2.0) for the molds. Overall, data upon opening indicated that both silages were well stored and presented a typical core microbiome.

During exposure to air, the temperature pattern of the Control silage was typical, with a two‐phase profile in the temperature curve, as also reported by Merry and Davies (1999). These authors described the first peak corresponding to an increase in yeast and AAB, and the second peak corresponding to the growth of molds. In our study, the change in metabolites and microbiota corresponded to a sharp drop in lactic acid, dominance of Saccharomycetales and more specifically an increase in OTUs linked to *Issatchenkia* in the first days after opening. The growth of *Issatchankia* was followed by an increase in the relative abundance of *Pichia*, *Acetobacter*, *Bacillus*, and *Paenibacillus*. It should be noted that the phenotypic classification of yeast species is often difficult due to switching between the teleomorph and anamorphic states (Kurtzman & Robnett, 2003). This may have introduced classification errors in the ITS database as already reported for *Candida krusei*, *Issatchenkia orientalis*, *Candida glycerinogenes*, and *Pichia kudriavzevii* that were shown to belong to the same species (Douglass et al., 2018), potentially explaining why the number of observed OTUs related to the *Issatchenkia* genus was high in our study. In all cases, all the microorganisms we detected—bacterial and fungal—can use lactic acid, as supported by the decrease in lactic acid concentrations along with an increase in pH. More specifically, the growth of *Acetobacter* could occur when yeasts are outnumbered or impaired by the accumulation of propionic acid (Dolci et al., 2011). This genus can convert ethanol into acetic acid in the presence of oxygen (Mamlouk & Gullo, 2013). It can also oxidize lactate and acetate into carbon dioxide and water (Pahlow et al., 2003). The presence of ethanol was observed since its concentration decreased more than 20 times from the highest value measured on day 0 to its lowest value after 10 days in the control, while it was reduced only by half in LB + LH. More than half the ethanol measured on day 0 was detected around day 5 and 6 when the peak temperature was reached. These modifications were followed by the growth of *Penicillium*‐related OTUs. The observed changes in pH, lactic acid, acetic acid, and ethanol were thus fully explained by the succession of different bacterial and fungal OTUs (Figure 1).

Few studies testing the microbial dynamic following aerobic exposure of silage have been published. When testing whole‐plant corn silage, Hu et al. (2018) showed that the abundance of *Sporolactobacillus* increased sharply after 72 h of exposure to air, but fungal diversity was not tested in their study. In an assay performed on small grain silage, Dunière et al. (2017) observed that Bacillales and Lactobacillales were part of the core microbiome after 14 days of exposure to air. Also, Saccharomycetales constituted 70% of the fungal population, and most OTUs were assigned to *Kazhachstania* and *Pichia*. These observations confirm the importance of lactate‐assimilating yeast during aerobic deterioration. Still, the correlation between yeast counts after fermentation and aerobic stability of different silages is not yet clear. The specific genera of yeast could be involved, but changes in the bacterial population could also act as a precursor of shifts in the yeast population and numbers. Among bacteria, *Acetobacter* seems to be of interest. Indeed, Dolci et al. (2011) demonstrated that *Acetobacter* was present after five days of exposure in corn silage under polyethylene treatment, and *Kazachstania* (day 7) was observed before *Pichia* (day 9). Similarly, high abundances of *Acetobacter* and *Lactobacillus* have been reported in whole‐plant maize silage exposed to oxygen for seven days (Li & Nishino, 2013). The same authors noticed that the presence of *Acetobacter* was specific to corn silage and that the decrease in *Acetobacter* was concomitant with an increase in the abundance of *Pediococcus*. Interestingly, we also observed this relationship between the presence of *Pediococcus* and a lower abundance of *Acetobacter* in treated silage. Overall, our results on microbiota are in agreement with those obtained in previous studies in which non‐inoculated silage was exposed to oxygen. However, inoculated and non‐inoculated silages were not compared in any of the above‐mentioned studies.

Inoculation with *L. buchneri* and *L. hilgardii* significantly improved the AS of bag silage, a result consistent with published data (Ferrero, Piano, et al., 2019; Nair et al., 2019). The temperature started to increase only on day 6 after opening without the two successive peaks observed in the Control silage. Interestingly, the lactic acid concentration remained stable with time, resulting in higher stability after silage opening, as suggested by the pH ranged between 3.97 and 4.10 during exposure to air. Similarly, the concentrations of acetic acid (4.4–9.9 g kg^−1^ DM) and ethanol (3.4–3.8 g kg^−1^ DM) remained in the same range during the 10 days of exposure to air. As a result, the lactic:total VFA ratio did not decrease in the LB + LH silage, whereas it decreased dramatically to reach 0.3 in the Control silage. In addition to these observations on the fermentation parameters, differences in microbiota can be summarized as a higher diversity of OTUs for both the bacterial and fungal microbiota, a more dynamic population of LAB, and a non‐lactate consuming yeast population (Figures 2, 3, 4, 5). Indeed, the abundance of *Lactobacillus* was more than 78% in the first eight days and was still 57% on day 10. This higher abundance of *Lactobacillus* might have contributed to the biocontrol of undesirable microorganisms (Castellano et al., 2017; Li et al., 2015). Remarkably, the presence of *Acetobacter* was observed in only one of the three replications using the LB + LH silage and was accompanied by higher temperatures during AS, thereby explaining the higher inter‐individual variation from day 8 on in Figure 1. In the same way, other undesirable bacteria belonging to the *Bacillales* order were less abundant in the LB + LH silage. Regarding the fungal population, the observed changes during the 10 days of exposure to air began with a higher diversity of ITS amplicons, the early disappearance of *Issatchenkia*, a lower abundance of the yeast *Pichia*, *Wickerhamomyces*, all of which are lactate‐assimilating yeasts (Kasmaei et al., 2017). This trend enabled a higher proportion of *Saccharomyces*‐related OTUs during the AS assay (Dunière et al., 2015). Overall, silage inoculation allowed a switch from *Pichia*‐*Issatchenkia* to *Saccharomyces*‐related yeast OTUs. All observed changes in the fermentation parameters were fully explained by the succession of the different bacterial and fungal OTUs. This study is the first to highlight how silage inoculation with *L. buchneri* and *L. hilgardii* improves aerobic stability thanks to maintaining higher microbial diversity and LAB fitness upon opening, avoiding the domination of a few yeasts and opportunistic facultative anaerobe bacteria that can use lactic acid (Figure 1).

Mycotoxins are secondary metabolites secreted by fungal organisms mostly belonging to the genera *Aspergillus*, *Fusarium*, *Alternaria*, and *Penicillium*. They have toxic effects on animals and may reduce feed intake and hence performance while inducing inflammatory status. Mycotoxins are not widely studied in silage, even though silage represents a major part of the diet of ruminants. The total amount of mycotoxins ingested by cows may be greater than the maximum concentrations allowed or recommended in ruminant diets by the US FDA or the EU. Different strategies to limit the production of mycotoxins in silage during the field, pre‐harvest, harvest, and ensiling phases exist and were recently reviewed (Ogunade et al., 2018). These strategies included the use of microbial inoculants to insure good ensiling conditions and to improve aerobic stability. One objective of our study was to investigate if the change in bacterial and fungal populations thanks to silage inoculation could influence mycotoxin content during the opening phase. Upon opening, three mycotoxins were detected, namely, fumonisin, beauvericin, and zearalenone (ZEA). Their presence at low levels confirmed good ensiling conditions.

During the opening period, concentrations of FB1 fluctuated with time but no particular trend was identified, while FB_2_/B_3_ concentrations remained low in all samples. The concentration of ZEA, mainly produced by *F. graminearum* in corn remained constant over the 10‐day AS period and below the European Commission guidance level for ruminant feed, which is 0.5 mg kg^−1^ (2006/576/EC). The levels of beauvericin, a toxin produced by *Fusarium* and considered as an emerging mycotoxin, were similar to or higher than the reported maximum concentration (Reisinger et al., 2019). All these *Fusarium* metabolites are considered to be “pre‐harvest” metabolites and are not produced during the ensilage process or the feed‐out stage. DON mycotoxin was not detected upon opening but appeared during the AS trial, particularly in the Control silage. DON is also a “pre‐harvest” mycotoxin that is produced mainly by *F. graminearum* in corn, which also biosynthesizes ZEA. An interesting observation was the increase in DON in contrast to the relatively constant concentration of ZEA, and to the low abundance of *Fusarium* related OTUs. Contamination by *Fusarium* may increase later in the aerobic deterioration process, as observed previously (Borreani et al., 2005). It is not clear whether the observed increase in the concentration of DON is the result of fungal activity occurring within the ensiled material or is rather the result of the conversion of DON‐conjugates and glycosides into DON *in plantae* (Castellano et al., 2017). The presence of DON glycosides in the samples suggests that the *in plantae* conversion metabolism is active. Unlike *Fusarium* metabolites that are generally considered to be “pre‐harvest” in origin, roquefortine C is produced by *Penicillium* and is considered a mostly “post‐harvest” mycotoxin (Sumarah et al., 2005). Remarkably, the increase in roquefortine C up to 1.3 mg kg^−1^ coincided with the increase in the abundance of *Penicillium* in the Control silages in the final days of AS.

Inoculating silage with LB and LH positively affected concentrations of FB1, DON, and roquefortine C. Indeed, we observed a significant decrease in the concentration of FB1 during the AS assay from inoculation. As the concentration of FB1 decreased with time during the AS trial, it is possible that one of the microorganisms present mitigated this toxin, as proposed by Martinez Tuppia et al. (2017). The presence of oxygen could have provided an ideal environment for the degradation of the toxins since several of the enzymes involved require oxidative conditions (Burgess et al., 2016). We suggest that LB + LH favors a microorganism with mycotoxin‐degrading ability. In the same way, DON and roquefortine C were almost not detected in the LB + LH silages during the current AS trial. Only a few studies have tested the effects of microbial inoculants on mycotoxin degradation, especially during exposure to air. Even if the conclusions differ from one study to another one, it appears that the use of microbial inoculants can reduce concentrations of mycotoxins or prevent the growth of toxigenic fungi (Kung et al., 2003). This study is the first to suggest that inoculating silage with LB + LH could improve the safety of silage following exposure to air. Further work is now required to understand these mechanisms.

## 5 CONCLUSION

To conclude, our study confirmed that exposure to air increases results in a switch in both the composition and in the activity of fungal and bacterial communities. It also highlighted the fact that the use of *L. buchneri* in combination with *L. hilgardii* modified the consequences of exposure to air by maintaining higher microbial diversity after the first 24 h of opening, avoiding the dominance of a few bacteria and fungi that could be detrimental to the silage quality. The combination of LB and LH could thus be used not only to ensure good conditions during ensiling but also to reduce microbial spoilage during exposure to air. We expect that performing a similar trial at an earlier stage of fermentation would result in even greater differences in microbial diversity in comparison with untreated silage. It will be interesting to go further in our research to integrate all data from harvest to feed‐out, and, ultimately, to test the impact of the inoculants on the nutritional quality of silage and the zootechnical performance of cattle. It would also be interesting to study the relationships between epiphytic microbiota and the production of mycotoxins in the field and at harvest, the kinetic evolution of these parameters during ensiling, and their impact during the exposure to air, with or without inoculants.

## CONFLICT OF INTEREST

This study received funding from Lallemand SAS. Emmanuelle Apper is employed by Lallemand SAS, Pascal Drouin is employed by Lallemand Specialities. This does not alter the authors' adherence to all the journal policies on sharing data and materials.

## AUTHOR CONTRIBUTIONS

**Pascal Drouin:** Conceptualization (lead); formal analysis (lead); investigation (lead); methodology (lead); supervision (equal); writing‐original draft (lead). **Julien Tremblay:** Data curation (lead); formal analysis (supporting); methodology (supporting); validation (supporting); writing‐review & editing (supporting). **Justin Renaud:** Investigation (supporting); methodology (supporting); validation (supporting); writing‐review & editing (supporting). **E. Apper:** Project administration (lead); supervision (equal); validation (equal); writing‐review & editing (equal).

## ETHICS STATEMENT

None required.

## Data Availability

All data are provided in full in the paper except for the 16S and ITS rDNA raw reads from the microbiota analyses that have been deposited at the NCBI repository under BioProject accession number PRJNA595554: https://www.ncbi.nlm.nih.gov/bioproject/PRJNA595554.

## Footnotes

1
^b^Statistical results in relation to the time after opening for each parameter.

## APPENDIX A

**TABLE A1:** Read count summary for 16S and ITS amplicons

16S amplicons		ITS amplicons	
total_reads	7,664,868	total_reads	6,131,578
contaminants_reads	298	contaminants_reads	142,378
phix_reads	280	phix_reads	1988
non_contam_non_phix_reads	7,664,290	non_contam_non_phix_reads	5,987212
non_contam_non_phix_reads_1	3,568,887	non_contam_non_phix_reads_1	2,993,606
non_contam_non_phix_reads_2	3,568,887	non_contam_non_phix_reads_2	2,993,606
assembled_reads	3,529,364	assembled_reads	2,312,096
assembled_reads_QC_passed	3,487,145	assembled_reads_QC_passed	2,227,585

Cluster counts for assembled/clustered			Cluster counts for assembled/clustered		
#name	count	perc	#name	count	perc
2382	92,230	1.29%	2413	94,812	1.58%
2376	81,402	1.14%	2428	57,014	0.95%
2403	153,212	2.15%	2431	76,508	1.28%
2375	73,670	1.03%	2397	138,902	2.32%
2411	66,114	0.93%	2197	19,824	0.33%
2422	93,646	1.31%	2410	83,830	1.40%
2429	47,454	0.66%	2195	83,194	1.39%
2408	91,182	1.28%	2406	97,268	1.62%
2426	116,818	1.64%	2380	92,808	1.55%
2413	132,620	1.86%	2407	173,452	2.90%
2385	99,788	1.40%	2425	75,260	1.26%
2416	93,674	1.31%	2432	72,338	1.21%
2418	87,218	1.22%	2424	72,056	1.20%
2438	88,942	1.25%	2423	49,970	0.83%
2387	58,268	0.82%	2416	51,804	0.87%
2433	76,816	1.08%	2376	12,578	0.21%
2377	38,254	0.54%	2398	103,134	1.72%
2381	59,308	0.83%	2395	126,008	2.10%
2197	75,180	1.05%	2394	138,414	2.31%
2389	172,150	2.41%	2402	118,316	1.98%
2402	144,114	2.02%	2419	80,288	1.34%
2424	169,890	2.38%	2420	58,196	0.97%
2380	126,406	1.77%	2396	128,934	2.15%
2419	42,960	0.60%	2427	116,494	1.95%
2439	85,702	1.20%	2429	54,822	0.92%
2401	144,430	2.02%	2403	173,354	2.90%
2420	100,232	1.40%	2434	85,496	1.43%
2198	75,344	1.06%	2439	98,916	1.65%
2423	64,358	0.90%	2409	136,730	2.28%
2421	108,046	1.51%	2199	34,884	0.58%
2400	129,260	1.81%	2414	82,380	1.38%
2388	134,130	1.88%	2392	92,258	1.54%
2435	57,036	0.80%	2378	18,732	0.31%
2383	70,706	0.99%	2426	40,244	0.67%
2199	48,140	0.67%	2377	47,356	0.79%
2379	81,448	1.14%	2391	163,534	2.73%
2407	85,432	1.20%	2388	89,880	1.50%
2412	118,182	1.66%	2408	110,552	1.85%
2391	114,516	1.60%	2422	68,370	1.14%
2436	85,620	1.20%	2379	28,812	0.48%
2431	24,546	0.34%	2412	78,770	1.32%
2195	144,402	2.02%	2436	74,368	1.24%
2396	199,538	2.80%	2375	51,428	0.86%
2427	135,650	1.90%	2198	9002	0.15%
2425	171,694	2.41%	2387	60,360	1.01%
2415	80,166	1.12%	2390	120,674	2.02%
2434	62,878	0.88%	2389	80,820	1.35%
2437	108,170	1.52%	2386	88,460	1.48%
2397	161,404	2.26%	2435	63,960	1.07%
2393	76,564	1.07%	2430	87,180	1.46%
2404	110,324	1.55%	2382	115,470	1.93%
2390	139,698	1.96%	2433	79,832	1.33%
2395	57,804	0.81%	2404	82,782	1.38%
2430	85,650	1.20%	2400	107,926	1.80%
2432	74,346	1.04%	2411	77,572	1.30%
2406	144,886	2.03%	2399	131,086	2.19%
2384	98,952	1.39%	2421	76,712	1.28%
2378	111,712	1.57%	2401	77,728	1.30%
2414	137,914	1.93%	2393	82,994	1.39%
2386	101,126	1.42%	2385	108,970	1.82%
2410	98,530	1.38%	2418	70,704	1.18%
2417	56,276	0.79%	2381	65,316	1.09%
2398	161,706	2.27%	2196	117,000	1.95%
2392	136,352	1.91%	2417	55,586	0.93%
2409	120,248	1.68%	2405	94,538	1.58%
2394	142,988	2.00%	2415	109,506	1.83%
2405	114,394	1.60%	2438	75,154	1.26%
2399	115,196	1.61%	2437	76,582	1.28%
2428	99,088	1.39%	2384	100,432	1.68%
2196	81,674	1.14%	2383	118,578	1.98%
Clustered	3,362,460 sequences give a total of 574 clusters		Clustered	1,481,801 sequences give a total of 878 clusters	
Classifed clusters	3,362,460 sequences packed in 574 clusters		Classifed clusters	1,481,716 sequences packed in 862 clusters	

**TABLE A2:** Mycotoxins and Fungal metabolites screened by LC‐MS with data‐independent acquisition

Name	RT (min)	m/z	Name	RT (min)	m/z
4'.5‐bisdeoxy‐dothistromin	3.52	341.0656	Fumonisin B3	2.83	690.4059
15‐acetylDON	2.64	339.14381	Fusarenon‐X	2.48	355.1387
3.15‐aDON	3.16	381.15438	Fusarubin	3.15	307.0812
3.7.15‐triacetyl‐DON	3.31	423.16494	Griseofulvin	3.28	353.0786
3‐acetylDON	2.71	339.14381	Helminthosporin	4.31	271.0601
5‐hydroxy culmorin	2.57	237.18491	HT‐2 Toxin	3.11	425.217
AAL toxin TB	2.71	506.33234	Isomarticin	3.13	377.0867
Aflatoxin B1	3.07	313.07065	iso‐Roridine E	3.74	537.2432
Aflatoxin B2	2.99	315.0863	Koninginin A	3.45	285.206
Aflatoxin G1	2.99	329.06556	Koninginin E	3.4	283.0612
Aflatoxin G2	2.9	331.08121	Kotanin	3.52	439.1387
Aflatoxin M1	2.79	329.06556	Macrosporin	3.76	285.0757
Alantrypinone	2.82	373.1295	Marticin	3.23	515.2639
Altenuene	2.89	293.10195	Meleagrin	2.73	434.1823
Alternariol	3.15	259.06008	Mellein	3.4	179.0703
Alternariol methyl ether	3.57	273.07573	Neosolaniol	2.52	405.1497
Altersolanol A	2.47	337.09178	Nivalenol	2.13	313.1282
Atranone B	3.71	447.23771	Ochratoxin A	3.54	404.0895
Averufin	4.24	369.09686	Penicillin V	3.1	351.1009
Beauvericin	4.49	806.39368	Penitrem A	4.18	634.293
Bostrycin	2.54	337.09178	Phacidin	3.67	295.154
Brevianamide A	2.91	366.1812	Pyrenopherol	2.70	335.1449
Cercosporin	3.57	535.1599	Roquefortine C	2.81	390.1924
Chaetoglobulosin A	3.61	529.26968	Roridin A	3.41	555.2537
Chaetoglobulosin C	3.75	529.26968	Rugulosin	3.69	543.1286
Chaetoviridin A	4.13	433.14123	Satratoxin F	3.40	565.203
Citrinin	3.35	251.09138	Satratoxin G	3.16	567.2175
Cryptosporiopsin	3.26	265.00287	Satratoxin H	2.87	545.2381
Culmorin	3.49	239.20054	Stachybocin B	4.00	899.5052
Deoxynivalenol	2.28	297.13325	Stachybocin C	4.01	899.5052
Diacetoxyscirpenol	3.05	367.17511	Sterigmatocystin	3.7	325.0707
DON‐glucoside	2.19	481.1659	stipitatic acid	2.296	183.0288
Emodin	3.78	271.0601	T‐2 tetraol	2.09	299.1489
Enniatin A1	4.63	690.4300	T‐2 Toxin	3.46	489.207
Enniatin B	4.41	662.39539	traversianal	4.26	317.2111
Enniatin B1	4.52	676.41034	Trichoverrol A	2.9	443.2017
Ergocorninine	2.77	562.30238	Verrucarin J	3.91	485.217
Ergocristinine	2.90	610.30238	Verrucarol	2.48	267.1591
Ergocryptinine	2.83	576.31803	Viridicatin	3.28	238.0862
Frequintin	3.41	253.14342	Walleminone	3.4	253.1798
Fumonisin B1	2.72	722.39573	Zearalenone	3.4	319.154
Fumonisin B2	2.89	706.40082	α‐cyclopiazonic acid	3.84	337.1547

**TABLE A3:** Tested fungal metabolites specific to *Penicillium*, *Fusarium*, and *Alternaria*

Detected compound	Formula	Precursor m/z	RT (min)	MassBank Accension^a^	Produced by: (genus)
Beauvericin	C_45_H_57_N_3_O_9_	806.3937	*4.49*	AC000574‐AC000582	*Fusarium/Beauveria*
Enniatin A_1_	C_35_H_61_N_3_O_9_	690.4300	*4.63*	AC000435‐AC000443	*Fusarium*
Enniatin B_1_	C_34_H_59_N_3_O_9_	676.4103	4.52	AC000462‐AC000470	*Fusarium*
Enniatin B	C_33_H_57_N_3_O_9_	662.3954	4.41	AC000453‐AC000461	*Fusarium*
Fumonisin B_1_	C_34_H_59_NO_15_	722.3957	2.72	AC000471‐AC000479	*Fusarium*
Fumonisin B_2_/B_3_	C_34_H_59_NO_14_	706.4008	*2.83/2.89*	AC000480‐AC000488	*Fusarium*
Acetyldeoxynivalenol	C_17_H_22_O_7_	339.1438	2.64	AC000372‐ AC000391	*Fusarium*
Deoxynivalenol	C_15_H_20_O_6_	297.1333	2.28	AC000110‐ AC000114	*Fusarium*
Zearalenone	C_18_H_22_O_5_	319.1540	3.4	AC000288‐ AC000292	*Fusarium*
Roquefortine C	C_22_H_23_N_5_O_2_	390.1924	2.81	AC000214‐AC000223	*Penicillium*
Alternariol	C_14_H_10_O_5_	259.0601	3.15	AC000060‐AC000062	*Alternaria*
Alternariol monomethyl ether	C_15_H_12_O_5_	273.0757	3.57	AC000063‐AC000065	*Alternaria*

^a^ https://massbank.eu/MassBank/.
